# High Prevalence of New Clinically Significant Findings in Patients With Embolic Stroke of Unknown Source Evaluated by Cardiac Magnetic Resonance Imaging

**DOI:** 10.1161/JAHA.123.031489

**Published:** 2024-01-19

**Authors:** Irum D. Kotadia, Robert O'Dowling, Akosua Aboagye, Richard J. Crawley, Neil Bodagh, Ali Gharaviri, Daniel O'Hare, Jose Alonso Solis‐Lemus, Caroline H. Roney, Iain Sim, Deborah Ramsey, David Newby, Amedeo Chiribiri, Sven Plein, Laszlo Sztriha, Paul Scott, Pier‐Giorgio Masci, James Harrison, Michelle C. Williams, Jonathan Birns, Peter Somerville, Ajay Bhalla, Steven Niederer, Mark O'Neill, Steven E. Williams

**Affiliations:** ^1^ School of Biomedical Engineering and Imaging Sciences, King’s College London London United Kingdom; ^2^ Guy’s and St Thomas’ National Health Service Foundation Trust London United Kingdom; ^3^ King’s College Hospital London United Kingdom; ^4^ Princess Royal University Hospital London United Kingdom; ^5^ Centre for Cardiovascular Science, The University of Edinburgh Edinburgh United Kingdom

**Keywords:** cardiac magnetic resonance imaging, cardiovascular disease, embolic stroke of unknown source, incidental finding, ischemic stroke, Ischemic Stroke, Cerebrovascular Disease/Stroke, Atrial Fibrillation, Magnetic Resonance Imaging (MRI)

## Abstract

**Background:**

Embolic stroke of unknown source (ESUS) accounts for 1 in 6 ischemic strokes. Current guidelines do not recommend routine cardiac magnetic resonance (CMR) imaging in ESUS, and beyond the identification of cardioembolic sources, there are no data assessing new clinical findings from CMR in ESUS. This study aimed to assess the prevalence of new cardiac and noncardiac findings and to determine their impact on clinical care in patients with ESUS.

**Methods and Results:**

In this prospective, multicenter, observational study, CMR imaging was performed within 3 months of ESUS. All scans were reported according to standard clinical practice. A new clinical finding was defined as one not previously identified through prior clinical evaluation. A clinically significant finding was defined as one resulting in further investigation, follow‐up, or treatment. A change in patient care was defined as initiation of medical, interventional, surgical, or palliative care. From 102 patients recruited, 96 underwent CMR imaging. One or more new clinical findings were observed in 59 patients (61%). New findings were clinically significant in 48 (81%) of these patients. Of 40 patients with a new clinically significant cardiac finding, 21 (53%) experienced a change in care (medical therapy, n=15; interventional/surgical procedure, n=6). In 12 patients with a new clinically significant extracardiac finding, 6 (50%) experienced a change in care (medical therapy, n=4; palliative care, n=2).

**Conclusions:**

CMR imaging identifies new clinically significant cardiac and noncardiac findings in half of patients with recent ESUS. Advanced cardiovascular screening should be considered in patients with ESUS.

**Registration:**

URL: https://www.clinicaltrials.gov; Unique identifier: NCT04555538.

**Nonstandard Abbreviations and Acronyms**
AMISTAD: Asymptomatic Myocardial Ischaemia in Stroke and Atherosclerotic Disease
CARM‐AF: Atrial Cardiac Magnetic Resonance Imaging in Patients With Embolic Stroke of Unknown Source Without Documented Atrial Fibrillation
ESUS: embolic stroke of unknown source
MASS: Multiple Atherosclerosis Site in Stroke
SCOT‐HEART: Scottish Computed Tomography of the Heart

**Clinical Perspective**

**What Is New?**

- This is the first study to report the prevalence of cardiac and noncardiac findings on cardiac magnetic resonance imaging in patients following embolic stroke of unknown source.
- Half of patients presenting with embolic stroke of unknown source had a clinically significant finding on cardiac magnetic resonance imaging that required further investigation, follow‐up, or treatment.

**What Are the Clinical Implications?**

- Further research is required to determine patients with embolic stroke of unknown source who are most likely to benefit from advanced cardiovascular imaging.
- New clinically significant findings on cardiac magnetic resonance imaging may highlight patients who are at greater risk of future cardiovascular events and secondary stroke.

Clinical assessment to ascertain the underlying cause of stroke is imperative to reduce the risk of secondary stroke. Approximately 85% of all strokes are ischemic strokes, and 1 in 6 ischemic strokes are classified as an embolic stroke of unknown source (ESUS), following conventional clinical evaluation.^1^ Patients with ESUS are at higher risk of repeated stroke and future cardiovascular events compared with patients with nonembolic stroke.^2^, ^3^, ^4^

Several guidelines advocate cardiovascular imaging with transthoracic echocardiography to exclude a cardioembolic source in patients with ESUS. Standard of care does not routinely extend to advanced cardiovascular imaging (defined as cardiac magnetic resonance [CMR] imaging, transesophageal echocardiography, or cardiac computed tomography [CT]),^5^, ^6^ although the American Heart Association guidelines acknowledge that CMR imaging may provide additional information in a minority of patients with ESUS.^5^ These guidelines are evidenced by 1 small retrospective single‐center study aimed at evaluating cardiac causes of emboli in patients with ESUS.^7^ The guidelines do not currently guide patient selection for evaluation with advanced cardiovascular imaging.

In the few studies that have evaluated CMR imaging, it has proven to be a valuable tool for detection of cardioembolic sources in patients with ischemic stroke.^8^, ^9^ Although the frequency of incidental extracardiac findings in CMR imaging has been well documented in patients with cardiac disease^10^ or healthy volunteers,^11^ there are no data assessing new clinical findings using CMR imaging in patients following ESUS beyond assessment for a cardioembolic source.

The primary aim of this study is therefore to assess the prevalence of new clinically significant cardiac and extracardiac findings identified via CMR imaging in patients within 3 months of ESUS. The secondary aim was to describe the patient characteristics associated with the presence of clinically significant findings.

## METHODS

The CARM‐AF (Atrial Cardiac Magnetic Resonance Imaging in Patients With Embolic Stroke of Unknown Source Without Documented Atrial Fibrillation; ClinicalTrials.gov Identifier: NCT04555538) study is a prospective multicenter nonrandomized cohort study aiming to produce a CMR‐based predictive model to detect future atrial fibrillation in patients with embolic stroke of unknown source and elevated risk of cardiac structural abnormalities. Patient recruitment took place at Guy's and St Thomas' Hospital, King's College Hospital, and Princess Royal University Hospital. The full study design has been previously published.^12^ Ethical approval was granted by Health Research Authorities and the South London Research Ethics Committee (REC: 19//LO/1933). The study conforms to the principles outlined in the Declaration of Helsinki. The data that supports the findings of this study are available from the corresponding author on reasonable request.

### Patient Recruitment

Patients with CHA_2_DS_2_‐VASc score (congestive heart failure, hypertension, age 65–75 or ≥75 years [2 points], diabetes, stroke [2 points], vascular disease, female sex) ≥3 were identified in both inpatient and outpatient settings and recruited within 3 months of presentation with ESUS. Capacity to consent was assessed by the clinical team before recruitment. Eighty‐five patients provided written informed consent. When patients lacked capacity to consent, advice was sought from a consultee and assent obtained from the patient.

### Eligibility

All patients underwent a minimum set of investigations to confirm ESUS diagnosis. Eligibility was determined following brain imaging to confirm the diagnosis of ischemic stroke (CT or magnetic resonance imaging), vascular imaging of head and neck (CT angiography, magnetic resonance angiography, or carotid Doppler) to exclude carotid or cerebral artery stenosis, and heart rhythm monitoring (ECG and 24‐hour heart rhythm monitoring) to exclude atrial fibrillation. In keeping with the National Institute for Clinical Excellence guidelines, transthoracic echocardiography was performed in selected patients when deemed appropriate by the clinical team.^6^ Patients with high suspicion of patent foramen ovale or left ventricular thrombus underwent bubble or contrast echocardiography before recruitment. Diagnosis of ESUS was confirmed by an independent expert stroke physician after review of investigations. Detailed inclusion and exclusion criteria can be found in Table S1.

### Demographic Data and Patient Characteristics

Patient data, including age, sex, race, smoking history, alcohol intake, and medical history, were documented at the time of ESUS presentation. CHA_2_DS_2_‐VASc score was calculated following ESUS diagnosis, with all patients receiving at least 2 points for the presence of stroke. Body mass index was calculated for each patient, and weight category was assigned as healthy, overweight, obese (class 1 and 2), and obese (class 3), as per the National Institute of Clinical Excellence obesity classification.^13^ Further data pertaining to the index stroke, including clinical history, blood panel, and imaging investigations (carotid Doppler, transthoracic echocardiography, and brain CT or magnetic resonance imaging), were recorded. History of stroke was defined as a previous presentation with stroke symptoms and subsequent neuroradiology imaging confirmation. The presence of single or multiple acute embolic infarcts was determined by review of clinical neuroradiology imaging by expert neuroradiologists.

### CMR Imaging Protocol

CMR imaging was performed using a 1.5‐T MAGNETOM Aera MRI Scanner (Siemens Healthineers, Erlangen, Germany) equipped with an 18‐channel anterior body coil and a 32‐channel posterior spine coil. Localizers were acquired, followed by 2‐dimensional balanced steady‐state free procession multicardiac phase cine imaging at end expiration during a single breath‐hold. Short‐ and long‐axis cine imaging of the atria and ventricles was obtained, followed by dedicated 3‐dimensional atrial imaging after administration of gadolinium (Gadovist; Bayer Healthcare Pharmaceuticals, Berlin, Germany), at both early (magnetic resonance angiography) and late (20–30 minutes) time points. The complete CMR study protocol can be found in the study design.^12^

### Definition of New Clinical Findings

Clinical CMR reports were provided for all patients by an expert CMR imaging physician according to routine clinical practice. A new clinical finding was defined as one not previously documented in the medical records. New clinical findings were categorized as cardiac or extracardiac in origin. A new clinically significant finding was determined by the need for further investigation, follow‐up, or treatment. New clinically significant findings were not required to be directly attributable to the underlying cause of the index stroke. A change in patient care was defined as initiation of medical, interventional, surgical, or palliative treatment following clinical evaluation of a newly identified clinically significant finding. Classification of a new cardiomyopathy was performed by an expert clinician as part of routine clinical care following review of clinical history, imaging, and results of further investigations if clinically required. A focal myocardial infarct of potential embolic cause was determined radiographically by typical appearance on late gadolinium enhancement imaging of a small, discrete, punctate lesion with no corresponding regional wall motion abnormality.^14^

### Statistical Analysis

Statistical analyses were performed using RStudio, version 4.0.3. Normally distributed quantitative variables were presented as means and SDs. Nonnormally distributed data were presented as medians and interquartile ranges. Categorical variables were expressed as frequencies and percentages. Between‐group comparisons were made using χ^*2*^ or Fisher exact test for categorical variables and Student *t* test or Mann‐Whitney *U* test for continuous variables, as appropriate. Relative risks were calculated between groups. A 2‐tailed *P*<0.05 was considered statistically significant.

## RESULTS

**Figure 1:**
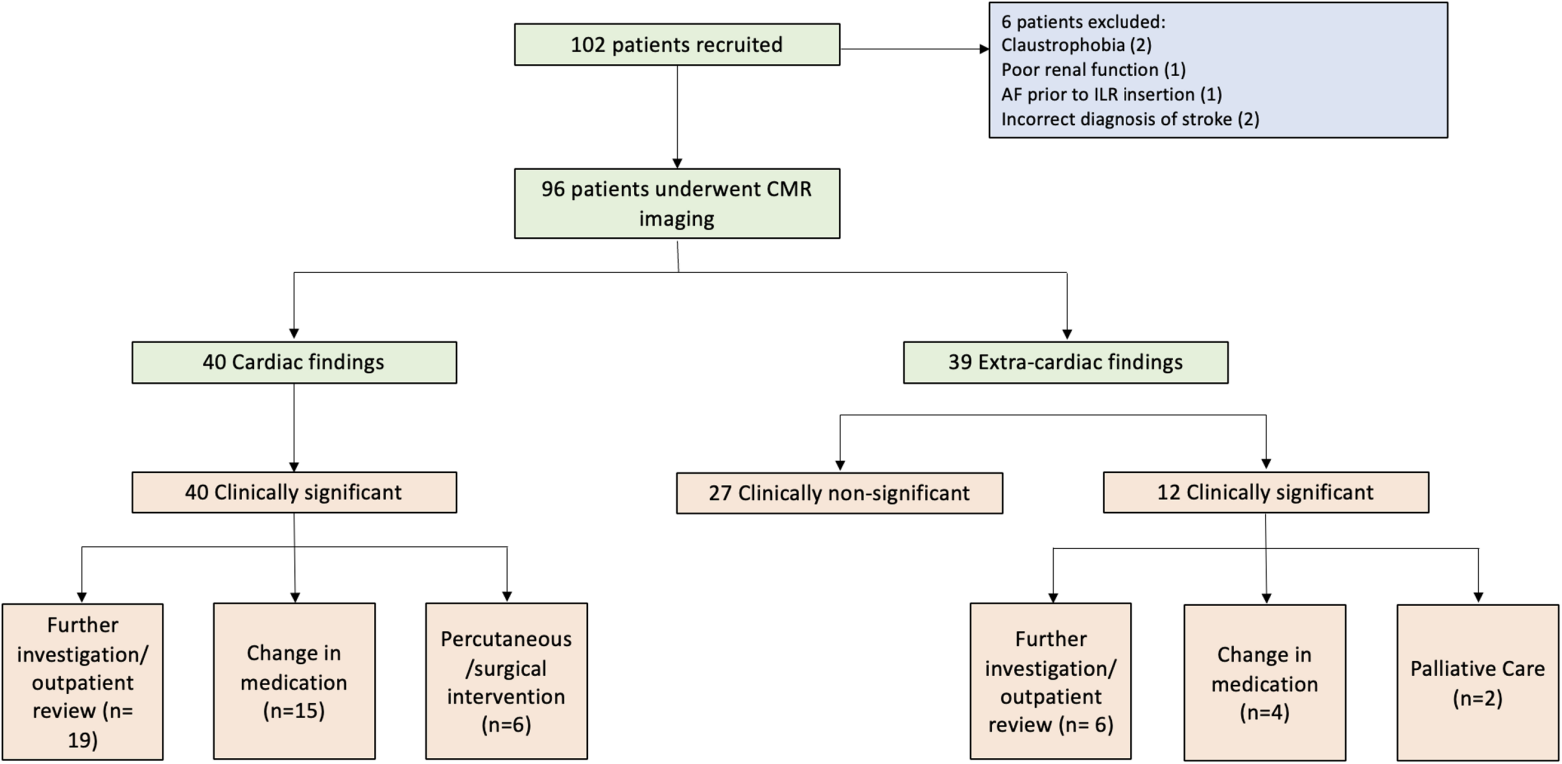
Patient recruitment. AF indicates atrial fibrillation; CMR, cardiac magnetic resonance; and ILR, implantable loop recorder.

Between September 2020 and September 2022, 102 patients were recruited to the study. Six patients were subsequently excluded from analysis. Four patients were excluded before CMR imaging because of claustrophobia, impaired renal function, or detection of atrial fibrillation subsequent to enrollment. Two patients were excluded following CMR imaging because of late diagnoses of brain abscess and brain metastases after clinical progression and further investigation (Figure 1). The remaining 96 patients were included in the analysis. The mean±SD age of included patients was 68±10 years, and 42% (n=40) were women (Table 1). Cause of ESUS was determined in only 1 patient following CMR imaging.

**Table 1:** Baseline Patient Characteristics Stratified by the Presence of Clinically Significant Findings

Patient characteristic	All patients	Patients with no clinically significant finding	Patients with a clinically significant finding	*P* value
Total No.	96	46	50	…
Age, mean±SD, y	68±10	68±10	68±10	0.802
Male sex, n (%)	56 (58)	25 (54.3)	31 (62)	0.572
BMI category, n (%)				0.036
Healthy	26 (27)	18 (40)	8 (16)	
Overweight	35 (37)	11 (24)	24 (48)	
Obese (class I/II)	30 (32)	14 (31)	16 (32)	
Morbidly obese (class III)	4 (4)	2 (4)	2 (4)	
Smoking status, n (%)				0.838
Nonsmoker	44 (54)	21 (53)	23 (56)	
Ex‐smoker	18 (22)	10 (25)	8 (20)	
Current smoker	19 (24)	9 (23)	10 (24)	
Unknown	15 (16)	6 (13)	9 (18)	
CHA_2_DS_2_‐VASc score, median±IQR	5±1	4±1	5±1	0.363
Hypertension, n (%)	68 (71)	29 (63)	39 (78)	0.166
Diabetes, n (%)	32 (33)	11 (24)	21 (42)	0.097
Peripheral vascular disease, n (%)	14 (15)	9 (20)	5 (10)	0.300
Heart failure, n (%)	3 (3)	1 (2)	2 (4)	1.000
Hypercholesterolemia, n (%)	17 (17)	8 (17)	8 (16)	1.000
Coronary artery disease, n (%)	10 (10)	5 (11)	5 (10)	1.000
Multiple acute brain infarcts, n (%)	44 (46)	26 (57)	18 (37)	0.084

BMI indicates body mass index; CHA_2_DS_2_‐VASc, congestive heart failure, hypertension, age 65–74 y, diabetes, stroke, vascular disease, age ≥75 y, sex; and IQR, interquartile range.

### New Clinical Findings

CMR imaging identified 79 new clinical findings in 59 (61%) patients. Sixteen patients (17%) had >1 new clinical finding reported (14 patients had 2 new clinical findings, 2 patients had 4 new clinical findings). In 48 patients, 52 new clinically significant findings were identified. In total, 36 patients (38%) had a clinically significant cardiac finding, 8 patients (8%) had a clinically significant extracardiac finding, and 4 patients (4%) had significant cardiac and extracardiac findings (Table 2). There were no statistically significant differences in demographic features or comorbidities between patients with and without clinically significant new clinical findings (Table 1). One patient (1%) had a new cause of stroke clearly identified.

**Table 2:** Distribution of New Clinical Findings on CMR Imaging in Patients With ESUS

Variable	No. of patients	% of Patients
All new findings	59	61
Clinically relevant finding	48	51
Cardiac	40	42
Extracardiac	12	13
Nonsignificant incidental extracardiac finding	22	23
Renal	9	9
Liver	5	5
Descending aorta	6	6
Gall stones	2	2
Thyroid	2	2
Other	3	3

CMR indicates cardiac magnetic resonance; and ESUS, embolic stroke of unknown source.

### Cardiac Findings

**Figure 2:**
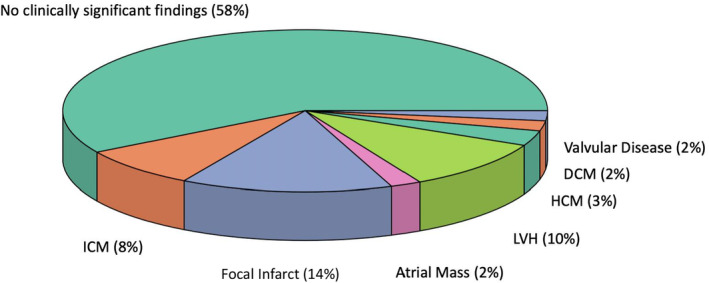
Clinically significant cardiac findings. DCM indicates dilated cardiomyopathy; HCM, hypertrophic cardiomyopathy; ICM, ischemic cardiomyopathy; and LVH, left ventricular hypertrophy.

**Figure 3:**
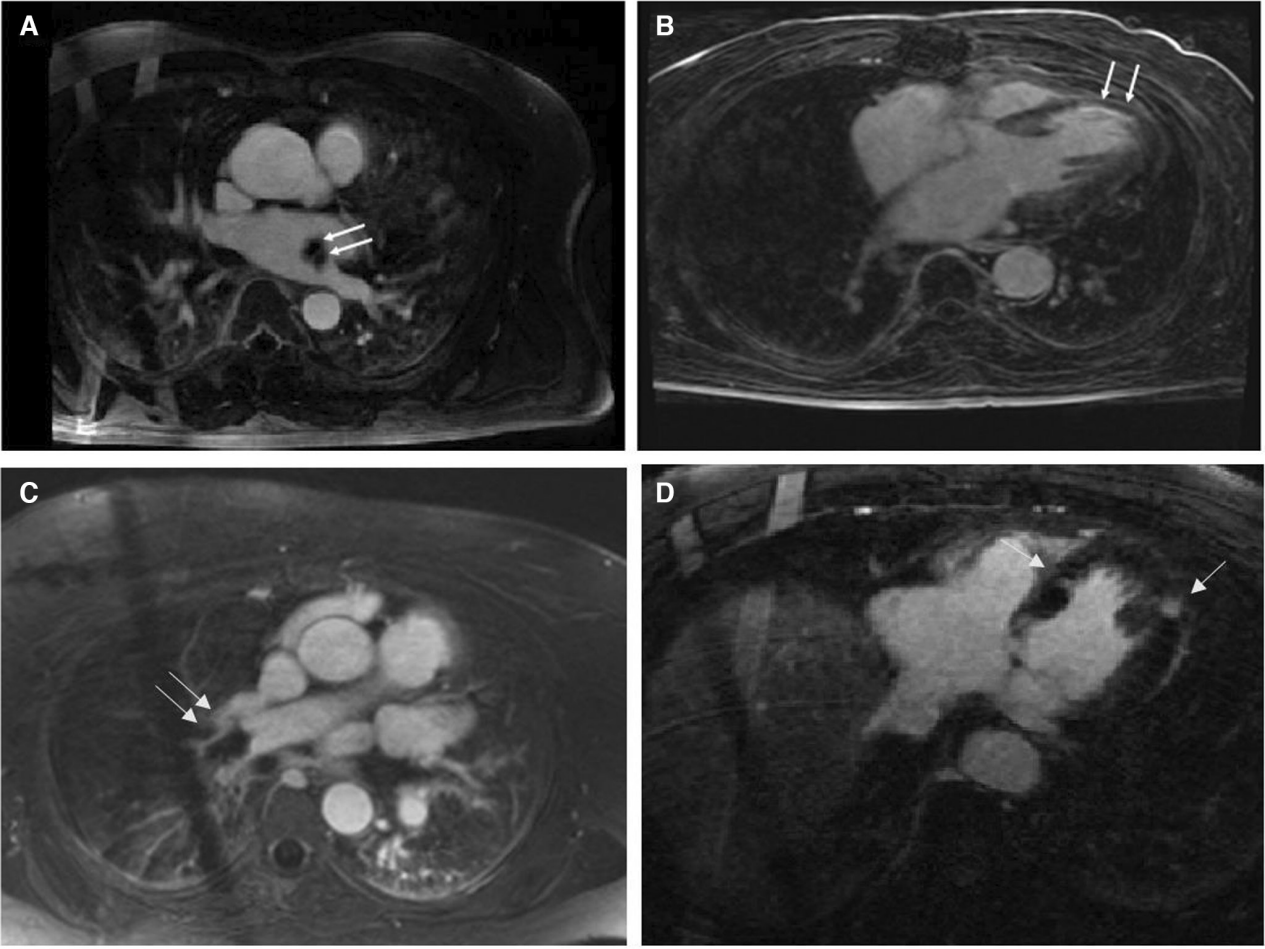
Examples of new clinically significant cardiac findings on cardiac magnetic resonance imaging. **A**, Contrast magnetic resonance angiogram (CMRA) taken 90 seconds after administration of gadolinium, showing left atrial mass (later confirmed on histology as atrial myxoma following surgical excision). **B**, Late gadolinium enhancement image taken 20 minutes after administration of contrast, showing thinned left ventricular myocardium with subendocardial enhancement consistent with previous myocardial infarction in the left anterior descending artery territory. Cine imaging confirmed severely reduced left ventricular function in keeping with a new diagnosis of ischemic cardiomyopathy. **C**, CMRA demonstrating large pulmonary embolus in the right pulmonary artery. **D**, Late gadolinium enhancement image showing multiple discrete infarcts with radiographical appearance of focal infarcts.

All cardiac findings were clinically significant. The most common cardiac findings were focal infarcts within the myocardium of the left ventricle (33%, n=13), left ventricular hypertrophy (25%, n=10), and ischemic cardiomyopathy (20%, n=8) (Figures 2 and 3). No patients were diagnosed with new left ventricular thrombus or patent foramen ovale. Detection of a new cardiac finding resulted in a change in patient care in 21 of 40 patients (53%). In 15 patients, additional medication was initiated. The remaining 6 patients underwent revascularization by percutaneous coronary intervention or cardiac surgery. New findings did not change management in patients already receiving optimal medical therapy. Two patients had a false‐positive CMR result through overestimation of the severity of valve disease and left ventricular dysfunction.

The prevalence of obesity was greater in patients with than without a cardiac finding (*P*=0.03; Table 3). There were no statistically significant differences in age, sex, smoking status, hypertension, diabetes, vascular disease, heart failure, or hypercholesterolemia between patients with and without a new clinically significant cardiac finding. No correlation was found between the presence of single or multiple acute infarcts and new clinically significant cardiac CMR findings.

**Table 3:** Characteristics of Patients With and Without Cardiac Findings

Patient characteristic	Patients with no cardiac finding	Patients with a cardiac finding	*P* value
Total No.	56	40	…
Age, mean±SD, y	69±10	68±10	0.619
Male sex, n (%)	32 (57)	24 (60)	0.944
BMI category, n (%)			0.030
Healthy	21 (38)	5 (130)	
Overweight	15 (27)	20 (50)	
Obese (class I/II)	17 (31)	13 (33)	
Morbidly obese (class III)	2 (4)	2 (5)	
Smoking status, n (%)			0.361
Nonsmoker	24 (49)	20 (63)	
Ex‐smoker	11 (22)	7 (22)	
Current smoker	14 (29)	5 (16)	
Unknown	7 (13)	8 (20)	
CHA_2_DS_2_‐VASc score, median±IQR	4±1	5±1	0.388
Hypertension, n (%)	36 (64)	32 (80)	0.149
Diabetes, n (%)	15 (27)	17 (43)	0.164
Peripheral vascular disease, n (%)	9 (16)	5 (13)	0.845
Heart failure, n (%)	1 (2)	2 (5)	0.766
Hypercholesterolemia, n (%)	10 (18)	6 (15)	0.926
Coronary artery disease, n (%)	5 (9)	5 (13)	0.821
Multiple acute brain infarcts, n (%)	33 (60)	11 (28)	0.300

BMI indicates body mass index; CHA_2_DS_2_‐VASc, congestive heart failure, hypertension, age 65–74 y, diabetes, stroke, vascular disease, age ≥75 y, sex; and IQR, interquartile range.

In addition to CMR imaging, 72 patients (75%) underwent echocardiography as part of routine clinical care. In keeping with the National Institute for Clinical Excellence guidelines, transthoracic echocardiography was performed in selected patients when deemed appropriate by the clinical team.^6^ Limited or poor views were documented in 27 patients (38%) because of body habitus or patient compliance. In 20 patients (28%), additional clinically significant findings were detected on CMR imaging. In 2 patients (3%), additional clinically significant findings were detected on echocardiography, both of which were valvular pathology. On stratification of patients with additional findings on CMR imaging compared with echocardiographic imaging, patients were more likely to have multiple acute brain infarcts (*P*=0.018). Although not reaching statistical significance, these patients also had a tendency to have a higher body mass index (*P*=0.052) (Table 4).

**Table 4:** Characteristics of Patients With Clinically Significant Cardiac Findings Stratified by Detection on Echocardiographic Imaging

Patient characteristic	Patients with no additional findings on MRI compared with echocardiography	Patients with additional findings on MRI compared with echocardiography	*P* value
Total No.	52	20	…
Age, mean±SD, y	69±10	66±8	0.279
Male sex, n (%)	29 (56)	12 (60)	0.953
BMI category, n (%)			0.052
Healthy	18 (35)	2 (10)	
Overweight	14 (18)	10 (50)	
Obese (class I/II)	18 (35)	6 (30)	
Morbidly obese (class III)	1 (2)	2 (10)	
Smoking status, n (%)			0.558
Nonsmoker	23 (44)	9 (45)	
Ex‐smoker	9 (17)	4 (20)	
Current smoker	12 (23)	2 (10)	
Unknown	8 (15)	8 (15)	
CHA_2_DS_2_‐VASc score, median±IQR	5±1	4±1	0.417
Hypertension, n (%)	38 (73)	13 (65)	0.700
Diabetes, n (%)	17 (32.7)	10 (50)	0.277
Peripheral vascular disease, n (%)	7 (14)	3 (15)	1.000
Heart failure, n (%)	2 (4)	1 (5)	1.000
Hypercholesterolemia, n (%)	7 (14)	5 (26)	0.416
Coronary artery disease, n (%)	5 (10)	3 (16)	0.803
Multiple acute brain infarcts, n (%)	31 (60)	5 (25)	0.018

BMI indicates body mass index; CHA_2_DS_2_‐VASc, congestive heart failure, hypertenion, age 65–74 y, diabetes, stroke, vascular disease, age ≥75 y, sex; IQR, interquartile range; and MRI, magnetic resonance imaging.

### Extracardiac Findings

Of 31 incidental extracardiac findings, 12 were clinically significant. The most common clinically significant extracardiac findings were lung malignancy (n=4, 4%, 2 primary malignancy and 2 pulmonary metastases), dilated ascending aorta (n=3, 3%), and pulmonary embolus (n=2, 2%) (Table 5). The most common nonsignificant extracardiac findings were renal cysts (n=9, 9%) and liver cysts (n=4, 4%). The finding of a clinically significant extracardiac finding changed patient management in 6 (50%) patients. In 2 patients with lung malignancy, the disease status was advanced and palliative care was offered. The remaining 2 patients underwent chemotherapy and/or radiotherapy. Of the patients diagnosed with pulmonary emboli, both received inpatient treatment, including anticoagulation.

**Table 5:** Clinically Significant Extracardiac Findings

Extracardiac finding	No. of patients (n=12)
Lung malignancy	4
Dilated ascending aorta	3
Pulmonary embolism	2
Liver cyst/hemangioma	1
Renal cyst	1
Thyroid nodule/cyst	1

There were no differences in patient characteristics or comorbidities between patients with and without a new extracardiac finding. No correlation was found between the presence of single or multiple acute infarcts and new extracardiac CMR findings (Table S2).

## DISCUSSION

This is the first study to examine the prevalence, impact, and associations of both cardiac and extracardiac new clinical findings from CMR imaging in patients with recent ESUS. The main findings are first that CMR imaging identifies new cardiac or extracardiac findings in almost two‐thirds of patients (61%) when performed within 3 months of presentation with ESUS. Second, most new findings are clinically significant, resulting in a change in patient care in approximately one‐third (28%) of patients with ESUS.

### Role of Cardiac Imaging in Patients With ESUS

Cardiac imaging in the context of acute ischemic stroke is currently performed for the detection of cardioembolic sources of ischemic stroke. However, there is considerable overlap between major risk factors for ischemic stroke and cardiovascular disease, including smoking, alcohol, obesity, hypertension, diabetes, and hypercholesterolemia. Although cardiac pathology may account for an increase in ischemic stroke risk, ischemic stroke may be the primary presentation of previously undetected cardiovascular disease that requires further investigation and management. To our knowledge, this study is the first to report the prevalence of cardiac and extracardiac findings identified via CMR imaging in patients with ESUS and its impact on subsequent clinical decision‐making and management.

The risk of future cardiovascular events in patients with ischemic stroke has previously been highlighted in a meta‐analysis determining the prevalence of asymptomatic coronary artery disease and incidence of myocardial infarction in patients following acute ischemic stroke.^15^ The authors reported an incidence of 3% of myocardial infarction within 1 year of an acute ischemic stroke and noted 1 in 3 patients had a coronary artery lesion of clinical significance (>50% stenosis). Further supporting evidence of cardiovascular risk in patients with ischemic stroke was published in the AMISTAD (Asymptomatic Myocardial Ischaemia in Stroke and Atherosclerotic Disease) study, which reported a 2‐year hazard ratio of 3.43 (95% CI, 1.48–7.93) of patients with ischemic stroke and a diagnosis of significant coronary artery stenosis (>50%) in ≥1 vessels,^16^ and the MASS (Multiple Atherosclerosis Site in Stroke) study, which demonstrated prevalence of atherosclerotic coronary plaque of 51% in patients with no evidence of plaque on cerebral imaging.^17^ In keeping with the strong evidence linking coronary artery disease and ischemic stroke, in this study, we found the most common clinically significant cardiac findings were ischemic cardiomyopathy, focal myocardial infarctions, and left ventricular hypertrophy, with over half of patients receiving additional medication or revascularization for risk reduction of future cardiovascular events. These findings highlight the need to identify patients presenting with ischemic stroke and undiagnosed cardiovascular disease, thereby facilitating the initiation of risk reduction therapies.

### Advanced Cardiovascular Imaging in Patients With ESUS

Current guidelines for the acute management of ischemic stroke recommend cardiac imaging using transthoracic echocardiography when appropriate.^5^ However, a prospective study of 548 patients undergoing transthoracic echocardiography found clinically significant changes requiring further investigation in 9% of patients, resulting in a change to patient care in only 5% of patients. This suggests a lower yield of clinically significant findings identified via transthoracic echocardiography when used unselectively in patients with ischemic stroke compared with CMR imaging in patients with ESUS, as we report in the present study.^18^ With a trend toward an elevated body mass index in this patient cohort, body habitus and patient compliance attributable to stroke severity may add further limitations to the utility of transthoracic echocardiography, as suggested by the current data.^19^

The additional value of advanced cardiovascular imaging in the detection of cardioembolic disease has been briefly described in the literature.^20^ A prospective observational study diagnosed left ventricular thrombus in 12 patients of 60 examined by CMR imaging. Only 1 patient had a left ventricular thrombus detected using transthoracic echocardiography (*P*=0.04).^8^ In patients with a history of myocardial infarction or reduced left ventricular function, a prediction model showed a net improvement of 0.46 (95% CI, 0.08–0.82; *P*=0.016) in cardioembolic stroke reclassification. Furthermore, a large retrospective analysis of 250 patients with ischemic stroke who underwent CMR imaging found 14 patients (5.6%) who required escalation of antithrombotic therapy from antiplatelets to full‐dose anticoagulation. Although these studies provide evidence supporting the use of CMR imaging to detect cardioembolic disease in patients with ESUS, they do not present data on the utility of CMR imaging for the identification of new clinically significant findings beyond left ventricular thrombus.

In addition to CMR imaging, transesophageal echocardiography and cardiac CT have also been described as methods for improving the clinical detection of cardiovascular disease in patients with ESUS. A small, prospective, single‐center study sought to use multidetector CT to perform a combined examination of the heart, aortic arch, and intracranial and extracranial arteries to improve causative workup of ischemic stroke. Results demonstrated good sensitivity (72%) and high specificity (95%) for detection of cardioembolic sources of stroke.^21^ However, multidetector CT failed to detect regional wall motion abnormality of the myocardium in 1 of 9 patients and 3 of 13 cases of septal abnormality when compared with transesophageal echocardiography. A further study of 137 patients noted an even higher sensitivity and specificity of 89% and 100%, respectively, but also failed to detect 5 of 22 patient foramen ovales and 3 of 11 atrial septal aneurysms that were detected on transesophageal echocardiography.^22^

In comparison, a meta‐analysis of patients undergoing transesophageal echocardiography following an ESUS would result in a change in therapeutic strategy in ≈1 in 7 patients.^23^ Although there are no studies comparing CT and CMR imaging in patients following ischemic stroke, a systematic review and meta‐analysis comparing transesophageal echocardiography with CMR imaging in identification of structural sources of emboli in patients with ischemic stroke found similar diagnostic yield between modalities, with the exception of patent foramen ovale (increased yield in transesophageal echocardiography) and left ventricular thrombus (increased yield in CMR).^24^ These findings were corroborated by a retrospective cohort study that found the diagnostic benefit of CMR imaging in detection of underlying stroke cause to be only slightly (>1%) improved compared with transesophageal echocardiography.^7^ Although our study did not draw comparisons with transesophageal echocardiography, 13 patients had focal myocardial infarcts of potential embolic cause during late gadolinium enhancement imaging with no evidence of left ventricular dysfunction. These findings are unlikely to have been detected on transesophageal echocardiography because of the differences in tissue characterization between modalities.

New clinical findings have also been shown to be important for patient management in other settings. A recent study of 2000 patients who underwent CMR imaging before radiofrequency ablation for atrial fibrillation found significant incidental findings in 8.6% of patients.^25^ Following clinical assessment, 42% of these patients did not undergo ablation. A substudy of the SCOT‐HEART (Scottish Computed Tomography of the Heart) trial, in which >4000 patients underwent CT coronary angiography, identified 10% of patients with clinically significant noncardiac findings, and in 3% of cases, these findings were the underlying cause of the patient's symptoms.^26^

### Impact of False‐Positive Clinically Significant Findings

Previous studies have explored the potential adverse impact of detecting incidental findings following CMR imaging in patients. In the UK Biobank data set, a systematic radiologist review of 1000 healthy participants resulted in 179 potentially serious incidental findings, of which a high proportion (88%) were characterized as false‐positive incidental findings following further clinical assessment.^11^ Patients reported adverse impacts of incidental findings on emotional well‐being (16.9%), finances (8.9%), and work or activities (5.6%). In addition, further clinical assessment in the form of outpatient attendance and investigations resulted in an increased burden of costs. However, this analysis was performed in healthy volunteers. In contrast, the present study found only 2 false‐positive clinically significant findings against the context of a much higher yield of true‐positive clinically significant findings in the ESUS cohort.

### Limitations

This study assesses the prevalence of new CMR findings in a specific cohort of patients with ESUS with at least 1 additional CHA_2_DS_2_‐VaSc risk factor (score, ≥3). It is unknown whether these findings generalize to patients with ESUS with lower cardiovascular risk. Although we report a high incidence of new clinically significant findings, and observe that CMR imaging informed changes in patient management, we do not present follow‐up data on the effects of these interventions on future cardiovascular outcomes or secondary stroke. A randomized controlled trial of routine CMR imaging in patients with ESUS would be required to address this question. Such a study would also allow a detailed cost‐effectiveness analysis to be performed.

CMR imaging was performed in patients within a 3‐month interval of the indexed stroke. It is unknown whether the findings reported in this study precede the index stroke or occurred within the 3‐month follow‐up period. These data do not provide an indication of whether early or delayed imaging would prove beneficial in this cohort of patients.

The present study detected only 1 diagnosis of cardioembolic disease of an atrial myxoma. Our detection rate of left ventricular thrombus and patent forman ovale is lower than described in the literature. This may be attributable to strict eligibility criteria and additional investigations performed before recruitment of patients with ESUS. A further contributing factor may be limitations of the CMR sequences performed as dedicated sequences to detect interatrial shunt were not performed.

Finally, other than increased obesity in patients with new cardiac clinical findings, we did not identify any patient characteristics identifying overall increased prevalence of new clinical findings. However, among patients with multiple brain infarcts, CMR imaging appeared to be of higher diagnostic value than transthoracic echocardiography imaging. Further evaluation is required to identify patient characteristics that may be used to optimize the choice of cardiovascular imaging modality in patients with ESUS.

## CONCLUSIONS

This study is the first to evaluate the prevalence, impact, and associations of cardiac and extracardiac new clinical findings from CMR imaging in patients with ESUS. Half of patients with ESUS with CHA_2_DS_2_‐VASc2 score ≥3 had a new clinically significant finding on CMR imaging. Of these patients, a significant number experienced a change in patient care. This study highlights the need for comprehensive cardiovascular evaluation in these patients, which may be achievable with CMR imaging. Further studies are required to identify patients with ESUS most likely to benefit from advanced cardiovascular imaging and to determine the impact of these changes on the future incidence of cardiovascular events and secondary stroke.

## Sources of Funding

This study was funded by the British Heart Foundation (PG/19/44/34368). Dr Kotadia acknowledges support from the British Heart Foundation (FS/CRTF/21/24166). Dr S. E. Williams acknowledges support from the British Heart Foundation (FS/20/26/34952). Professor Plein was funded by the British Heart Foundation under grant CH/16/2/32089. This work was supported by the Wellcome/EPSRC Centre for Medical Engineering (WT203148/Z/16/Z). Dr M. C. Williams is supported by the British Heart Foundation (FS/ICRF/20/26002). The authors acknowledge the support of the British Heart Foundation Centre for Research Excellence Award III (RE/18/5/34216).

## Disclosures

Professor O'Neill has received research support and honoraria from Biosense Webster and has received consultation fees from Medtronic, Biosense Webster, St. Jude/Abbott, and Siemens. Dr S. E. Williams has received research support from Biosense Webster, Abbott, and EPD‐Philips; and consultation fees from Imricor Medical Systems, LUMA Vision, and GSK. Dr M. C. Williams has given talks for Canon Medical Systems, Siemens Healthineers, and Novartis. The remaining authors have no disclosures to report.

## Supporting information

Tables S1–S2
