# Supplementary material for: High Prevalence of New Clinically Significant Findings in Patients With Embolic Stroke of Unknown Source Evaluated by Cardiac Magnetic Resonance Imaging
Source: J Am Heart Assoc. 2024 Jan 19;13(3):e031489. doi: 10.1161/JAHA.123.031489 (PMC11056130; doi:10.1161/JAHA.123.031489)
Supplement: Supplementary file 1 — Tables S1–S2 [file JAH3-13-e031489-s001.pdf]

SUPPLEMENTAL MATERIAL

Table S1. Inclusion and exclusion criteria for the CARM-AF study.

Inclusion Criteria	*Exclusion Criteria*
Acute ischaemic stroke confirmed on brain imaging within 3 months of study enrolment	History of atrial fibrillation
Patient able to provide consent or assent following consultation from next of kin	eGFR < 30ml/min
Expected survival greater than 12 months	Indication for pacemaker/implantable cardioverter-defibrillator
$CHA_2DS_2VASc_2$ score ≥ 3	Contra-indication to undergo cardiac MRI (eg. Severe claustrophobia, unable to lie flat for prolonged period)
Sinus rhythm on 12-lead-ECG, telemetery and/or 24 hour-monitor and a regular pulse on clinical examination	Carotid stenosis >50% on duplex ultrasound or CT angiography associated with anterior circulation infarct
Above 18 years of age	Vertebrobasilar stenosis >50% on CT/MR angiography associated with posterior circulation infarction
	Single, isolated lacunar infarct with a corresponding lacunar infarct on brain CT/MRI
	Specific aetiology for cause of stroke (eg. Arteritis, dissection, drug abuse)

eGFR = estimated glomerular filtration rate, MRI = magnetic resonance imaging, ECG = electrocardiogram, CT = computed tomography

Table S2. Characteristics of patients with and without incidental extra-cardiac findings.

**Patient Characteristic**	**No. of patients with no clinically significant extra-cardiac finding**	**No. of patients with a clinically significant extra-cardiac finding**	**P-value**
N =	64	32	
Age (mean±SD)	68±10	68±8	0.906
Male sex (%)	34 (53)	22 (69)	0.213
BMI category:			0.721
Healthy	17 (27)	9 (28)	
Overweight	22 (35)	13 (41)	
Obese (Class I/II)	22 (35)	8 (25)	
Morbidly obese (Class III)	2 (3)	2 (6)	
Smoking status:			0.560
Non-smoker	31 (56)	13 (50)	
Ex-smoker	13 (24)	5 (19)	
Current Smoker	11 (20)	8 (31)	
Unknown	9 (14)	6 (19)	
$CHADS_2VASc$ score (median ±IQR)	5±1	4±1	0.412
Hypertension (%)	43 (67)	25 (78)	0.383
Diabetes (%)	21 (33)	11 (34)	1.000
Peripheral vascular disease (%)	12 (19)	2 (6)	0.184
Heart Failure (%)	3 (5)	0 (0)	0.534
Hypercholesterolaemia (%)	11 (17)	5 (16)	1.000
Coronary artery disease (%)	9 (14)	1 (3)	0.194
Multiple acute brain infarcts (%)	29 (45)	15 (48)	0.950

SD = standard deviation, BMI = body mass index, IQR = interquartile range.
